# Supplementary material for: Plant diversity maintains multiple soil functions in future environments
Source: eLife. 2018 Nov 28;7:e41228. doi: 10.7554/eLife.41228 (PMC6296783; doi:10.7554/eLife.41228)
Supplement: Supplementary file 1. [file elife-41228-supp1.docx]

**Supplementary File 1**

Table S1. Plant species with the highest performance in monoculture for four soil response variables in four environmental contexts.

| Variable | Control | Elevated CO_2_ | Elevated Nitrogen (N) | Elevated CO_2_ + elevated N |
| --- | --- | --- | --- | --- |
| Root biomass | *S. scoparium* | *B. gracilis* | *P. villosum* | *A. repens* |
| Soil respiration | *B. gracilis* | *A. repens* | *A. repens* | *S. rigida* |
| Soil microbial biomass | *S. scoparium* | *S. rigida* | *L. capitata* | *S. rigida* |
| Soil aggregate stability | *A. gerardi* | *S. scoparium* | *L. capitata* | *A. gerardi* |
