# Supplementary material for: Plant diversity maintains multiple soil functions in future environments
Source: eLife. 2018 Nov 28;7:e41228. doi: 10.7554/eLife.41228 (PMC6296783; doi:10.7554/eLife.41228)
Supplement: Supplementary file 2. [file elife-41228-supp2.docx]

**Supplementary File 2**

Appendix A1: Code of general linear models.

proc glm data = *SOURCE.FILE*;

class co2 Nitrogen ring ;

model *RESPONSE.VARIABLE* = co2 ring(co2) Nitrogen

specrich

co2*Nitrogen

co2*specrich

Nitrogen*specrich

co2*Nitrogen*specrich

/ss3;

random ring(co2)/test;

run; quit;
